# Family carers' knowledge and practices in dementia care for hospitalized older adults: a multi-center cross-sectional study in Thailand

**DOI:** 10.3389/fmed.2025.1710146

**Published:** 2025-12-03

**Authors:** Kanthee Anantapong, Nahathai Wongpakaran, Suwanna Setthawatcharawanich, Sirinapa Aphisitphinyo, Nopdanai Sirimaharaj, Chatchawan Rattanabannakit, Pongsatorn Paholpak, Aimorn Jiraphan, Pakawat Wiwattanaworaset, Warut Aunjitsakul, Napakkawat Buathong, Pattharee Paholpak, Kritta Supanimitamorn, Kitikan Thana-udom, Pornjira Pariwatcharakul, Tinakon Wongpakaran, Huali Wang

**Affiliations:** 1 Department of Psychiatry, Faculty of Medicine, Prince of Songkla University, Hat Yai, Thailand; 2 Geriatric Psychiatry Unit, Department of Psychiatry, Faculty of Medicine, Chiang Mai University, Chiang Mai, Thailand; 3 Department of Internal Medicine, Faculty of Medicine, Prince of Songkla University, Hat Yai, Thailand; 4 Department of Psychiatry, Faculty of Medicine, Khon Kaen University, Khon Kaen, Thailand; 5 Neurology Division, Internal Medicine Faculty of Medicine, Chiang Mai University, Chiang Mai, Thailand; 6 Division of Neurology, Department of Internal Medicine, Faculty of Medicine, Siriraj Hospital, Mahidol University, Bangkok, Thailand; 7 Department of Family and Preventive Medicine, Faculty of Medicine, Prince of Songkla University, Hat Yai, Thailand; 8 Department of Psychiatry, Faculty of Medicine, Siriraj Hospital, Mahidol University, Bangkok, Thailand; 9 Dementia Care and Research Center, Peking University Institute of Mental Health, Beijing, China

**Keywords:** dementia care, family carers, hospitalization, Thailand, Dementia Knowledge Assessment Scale (DKAS-Thai)

## Abstract

**Background:**

Dementia-friendly hospital care is a growing priority in Thailand as the population ages, but the knowledge and experiences of family carers within well-resourced university hospitals remain underexplored—particularly regarding how care processes and cultural context affect outcomes.

**Methods:**

This multi-center cross-sectional study surveyed 136 family carers of older adults with dementia, recruited from outpatient clinics at four major university hospitals across Thailand. Data were collected via structured interviews assessing carer demographics, dementia knowledge using the DKAS-Thai scale, and carer experiences of hospital dementia care using the Implementation and Sufficiency of Dementia-Friendly Practices (ISDP) tool. Group comparisons and associations were explored using descriptive statistics and ANOVA.

**Results:**

Carers' knowledge about dementia was moderate overall, with higher scores among those with greater education and previous dementia exposure. Nevertheless, persistent gaps were noted, especially regarding the reversibility of dementia and behavioral management. While over 80% of carers reported adequate involvement in daily care and decision-making, the implementation of hospital dementia-friendly practices was inconsistent. Nearly half of respondents reported a lack of systematic dementia identification, limited individualized care planning, and insufficient discharge communication. Site-level differences and cultural influences, including filial obligation and low expectation of formal support, contributed to pronounced variability in carers' experiences. No significant linear association between DKAS-Thai and ISDP scores was observed, suggesting that knowledge alone may not translate into improved care experiences in complex, variable hospital settings. Carers highlighted insufficient communication, limited family participation in discharge planning, and gaps in advance care planning and palliative access, despite specialist hospital resources.

**Conclusions:**

Significant variability in dementia care implementation and knowledge persists across Thailand's university hospitals, highlighting the interplay of institutional practices and cultural factors. System-level interventions—such as standardized dementia screening, routine family involvement in multidisciplinary planning, and culturally responsive education for carers and staff—are recommended to bridge these gaps. These findings underscore the need for action not only in university hospitals but also in developing broader national dementia care strategies adapted to Thailand's social context.

## Introduction

Dementia is a progressive, debilitating syndrome that significantly impairs cognitive, functional, and psychosocial abilities, affecting millions of older adults worldwide (1). With increased longevity and changing demographic structures, dementia has emerged as one of the most pressing global public health challenges, especially for countries like Thailand with rapidly aging populations (2). According to the World Health Organization (WHO), over 55 million people globally are currently living with dementia, and this number is projected to almost double every 20 years, reaching 78 million in 2030 and 139 million by 2050. In Thailand, recent estimates suggest a continuing rise in dementia prevalence among older adults, representing a substantial burden both on healthcare systems and family networks (3).

In Thailand, the number of dementia cases was estimated at 670,047 in 2016, with projections rising markedly to 2,391,672 by 2050 according to the Global Burden of Disease Study 2019 (4). It is estimated that almost 90% of these individuals are cared for at home by family members, most commonly spouses or adult children, highlighting the critical role of families as the primary support system for PLWD in the country (5).

Family carers play a central role in the care trajectory of people living with dementia (PLWD), often providing the majority of day-to-day support and forming a crucial link between healthcare services and the individual with dementia (6). In the Thai cultural context—where family bonds and filial piety are highly valued—caring responsibilities typically fall to close relatives, most commonly daughters or spouses (7). This caregiving role is demanding and complex, encompassing emotional support, physical care, behavioral management, advocacy, and participation in treatment decisions. Family carers not only provide direct care but also navigate interactions with healthcare professionals, particularly during acute hospitalisations, which are common in the progression of dementia (8).

Older adults living with dementia are more likely to be admitted to general hospitals compared to their cognitively healthy counterparts, often for comorbid illnesses, falls, acute confusion (delirium), and other complications (9). Hospital environments, however, are rarely optimized for the unique care needs of PLWD—characterized by routine changes, unfamiliar surroundings, disjointed care, and time constraints on staff (10, 11). Hospitalization is a critical period that can exacerbate behavioral and psychological symptoms of dementia (BPSD), impair functional abilities, and cause distress both for PLWD and their family carers (12, 13). Numerous studies have reported that adverse outcomes, including prolonged hospital stays, functional decline, institutionalization, and mortality, are higher among hospitalized PLWD, underscoring the importance of dementia-appropriate care strategies (14).

Despite accumulating international evidence and guidelines advocating for person-centered dementia care in acute settings, implementation remains inconsistent, especially in low- and middle-income countries (LMICs) like Thailand. Gaps frequently exist in staff awareness and training, care coordination, effective communication, and inclusion of family carers as equal partners in care planning. Family carers' experiences, perspectives, and knowledge have a direct impact not only on the hospitalization outcomes for PLWD but also on their carers' psychological wellbeing, satisfaction, and confidence navigating the healthcare system (15, 16).

Systemic solutions to address these gaps have begun to emerge in Thailand, reflecting both policy-level initiatives and hospital-based strategies. At the national level, the Thai Ministry of Public Health has issued strategic plans aiming to integrate dementia care into primary healthcare services, improve early detection, and enhance carer support. Hospitals in major urban centers are increasingly implementing dementia-friendly practices, such as specialized wards, staff training in geriatric care, and environmental adaptations to reduce confusion and agitation among patients with dementia. Some institutions offer multi-disciplinary care teams, including geriatricians, psychiatrists, nurses, and social workers, to provide holistic assessments and continuity of care. Further, educational programs designed specifically for family carers have been piloted in collaboration with academic institutions, focusing on dementia awareness, behavioral management, and navigation of available community resources. Efforts are also being made to standardize protocols for the identification, documentation, and management of dementia within hospital electronic record systems. However, these approaches are not yet widespread and remain more accessible in tertiary hospitals than in smaller provincial or community settings. At the community level, networks of health volunteers and local clinics are being mobilized to conduct home visits, provide basic training, and facilitate referrals for people with dementia. Policy advocacy by non-governmental organizations has highlighted the need for expanded access to palliative and respite care services, as well as financial assistance for families affected by dementia-related disability.

The dementia knowledge of family carers is a key determinant of caregiving quality and satisfaction (6, 17). Greater understanding of dementia's manifestations, progression, and optimal management can empower carers, reduce misconceptions and stigma, and foster better cooperation with hospital staff (18). However, carer knowledge is influenced by multiple factors, including education, prior experience, sleep problems, depressed mood, loss of volition, chronic diseases, non-manual jobs, and contact with patients with dementia (19, 20).

In Thailand, limited research has examined the actual level of dementia knowledge among family carers, how this relates to their demographic characteristics, or how it translates to their experience and involvement within hospital settings.

Moreover, the extent to which hospitals in Thailand implement key elements of dementia-friendly care—such as staff awareness, adaptation of environments, personalisation of care, effective communication, continuity planning, and collaboration with family carers—has not been comprehensively studied from the carers' perspective. The quality of these processes can significantly shape carers' trust in the healthcare system, willingness to collaborate, and readiness to provide ongoing care after discharge (21, 22).

As hospitals are likely to see increasing numbers of PLWD in the coming decades, there is an urgent need to understand the current landscape of dementia care in acute settings from the viewpoint of those most intimately involved: the family carers. Assessing their knowledge levels, demographic characteristics, and the quality of their interaction and experience with hospital services can provide valuable guidance for targeted interventions, policy development, and resource allocation.

Although “knowledge about dementia” is fundamentally an individual-level characteristic, there are sound theoretical and practical reasons to expect a correlation with carers' perceptions of the implementation of dementia-friendly practices at the institutional level. Family carers with greater knowledge of dementia are often more aware of what constitutes best practice. They are thus better able to recognize, in both positive and negative ways, whether appropriate dementia-friendly measures are being implemented during a hospital stay. In the Thai healthcare context, knowledgeable carers may also communicate more effectively with healthcare staff, advocate for specialized needs, or request specific dementia-friendly adaptations, all of which could influence their perceptions and potentially prompt improvements in care delivery. Additionally, this understanding equips carers to provide more informed feedback, which may further encourage or support the institution's adoption of such practices. Therefore, exploring this correlation can yield valuable insights into how carer empowerment and education intersect with the experience and perception of care quality in university hospital settings.

Therefore, this study was designed to assess the knowledge and experiences of family carers of hospitalized older adults with dementia in Thailand, and to explore how carer knowledge relates to the implementation of dementia-friendly hospital care practices.

## Materials and methods

### Study design and setting

This study forms part of a larger multi-centered project titled “Knowledge and Practice of Family Carers and Professionals Regarding Dementia Care for Older Inpatients in General Hospitals: A Multi-Center, Cross-Sectional Study in Thailand.” This study employed a cross-sectional design and was conducted across four university hospitals in Thailand: Maharaj Nakorn Chiang Mai Hospital, Songklanagarind Hospital, Srinagarind Hospital, and Siriraj Hospital. These hospitals were purposively selected to represent geographically diverse regions (North, South, Northeast, and Central) and to ensure inclusion of leading tertiary care centers with established geriatric and neurological services for dementia care. The selection aimed to capture variations in hospital resources, protocols, and patient populations across different parts of the country. Inclusion criteria for hospital sites were: (1) status as a university-affiliated teaching hospital, (2) the presence of specialized inpatient services for older adults and dementia, and (3) agreement to participate in the multi-center study. Hospitals were excluded if they lacked acute inpatient wards for older adults or lacked a formally recognized dementia care program. Only a subset of the country's university hospitals was included, and the sample does not comprehensively represent all university hospitals in Thailand. The focus was on family carers of older adults with dementia who had been admitted to acute wards in general hospitals.

### Participants

Eligible participants were family members, relatives, or friends providing unpaid primary care to a person with dementia admitted to an acute hospital ward for at least 2 days within the previous year. Inclusion criteria for participants were: (1) age >18 years, (2) ability to communicate in Thai, (3) physically and mentally healthy as determined by clinical judgment, (4) identified as the primary carer during the index hospital admission. Exclusion criteria were: (1) paid/professional caregivers, (2) carers of patients who were not formally diagnosed with dementia by a physician, (3) those unable or unwilling to provide informed consent, and (4) individuals experiencing significant physical or cognitive impairment themselves. The minimum sample size required was 59 (α = 0.05, SD = 6.75, *d* = 0.05 × mean), but was increased to 120 (about 30–35 per site) to enhance psychometric analysis and consistency with broader study procedures.

### Recruitment procedures

This multicentre study was conducted in four university hospitals, each with multiple acute inpatient wards (e.g., Internal Medicine, Neurology, and Psychiatry wards) that routinely admit older adults with dementia experiencing acute medical, behavioral, or neuropsychiatric problems. University hospitals in Thailand are typically comprehensive tertiary care centers that incorporate acute care units, specialty inpatient wards, and outpatient clinics under a single institutional umbrella. Thus, the study population comprised individuals admitted to acute inpatient wards within these university hospitals. Potential participants were identified using outpatient lists from the Internal Medicine, Neurology, and Psychiatry departments. While these departments are primarily outpatient-based, they commonly follow and review patients who have recently been discharged from their respective acute inpatient (hospital) wards for post-discharge care and ongoing management. As such, outpatient clinic appointments were strategically used for recruitment, as this is the routine setting where family carers of previously hospitalized dementia patients are accessible following acute admissions.

Clinical staff external to the research team (e.g., nurses or nurse assistants) approached potential participants during their outpatient visits. The study was briefly introduced, and a Participant Information Sheet (PIS) was provided. Prospective participants were screened for eligibility (i.e., having cared for a dementia patient admitted to an acute hospital ward for at least 2 days in the prior year). Those meeting criteria and expressing interest were referred to by a research team member, who provided a comprehensive study explanation and obtained written informed consent.

### Data collection

After the consent process, individual interviews were conducted using a pre-determined, structured questionnaire. The interview covered demographic data, experiences regarding the care of their relatives with dementia during the most recent hospital admission, and general knowledge about dementia, assessed with the Dementia Knowledge Assessment Scale—Thai version (DKAS-Thai).

Interviews were conducted in a private, quiet room within the clinics by a research team member experienced in both clinical work and research. Each interview lasted approximately 15–30 min. During the interview process, research personnel coordinated with clinical teams to ensure family carers and their relatives would not lose their place in the clinic queue or miss medical appointments. All participants received compensation of 100 baht for their time. Upon request, participants were also provided with additional information and resources about dementia.

### Questionnaire and measurement

Two main instruments were used in this study:

- Dementia Knowledge Assessment Scale – Thai version (DKAS-Thai). Family carers' dementia knowledge was measured using the DKAS-Thai, a validated adaptation of the Dementia Knowledge Assessment Scale for Thai populations. The DKAS-Thai consists of 25 items covering topics such as dementia symptoms, risk factors, care considerations, and communication strategies. Responses are rated on a Likert-type scale (e.g., correct/incorrect/unsure), with higher total scores indicating a greater level of dementia knowledge. Previous research has demonstrated that the DKAS-Thai possesses good reliability and validity among Thai caregivers. The DKAS-Thai has demonstrated good internal consistency in prior studies, with a Cronbach's alpha of 0.825 (31).
- Implementation and Sufficiency of Dementia-Friendly Practices (ISDP). Hospital care processes were evaluated using a self-developed questionnaire entitled “Implementation and Sufficiency of Dementia-Friendly Practices.” This structured tool was developed based on current international guidelines and expert consensus for dementia care in acute settings. It includes 16 items assessing both the presence and perceived adequacy of key dementia-friendly practices across several domains, such as dementia identification, family involvement, communication, environmental personalization, and care planning. For each item, respondents indicated whether the practice was implemented as “No,” “Yes, but insufficient,” or “Yes, and sufficient.” Higher scores represent greater implementation and sufficiency of dementia-friendly approaches. The ISDP tool demonstrated acceptable internal consistency with a Cronbach's alpha of 0.88 in this study.

### Statistical analysis

All statistical analyses were performed using SPSS version 26 (IBM Corp., Armonk, NY, USA). Descriptive statistics—including means, standard deviations, frequencies, and percentages—were calculated to summarize demographic characteristics of family carers, caregiving profiles, and responses to items regarding dementia knowledge and hospital care experiences. Principal Component Analysis (PCA) with varimax rotation was used to examine the underlying structure of items related to the implementation and sufficiency of dementia-friendly practices (ISDP) in hospital settings. Components with eigenvalues greater than one were retained, and individual items with loadings above 0.40 were considered as significantly loading on each component. Group differences in dementia knowledge scores (DKAS-Thai) were evaluated using independent *t*-tests or one-way ANOVA for continuous, normally distributed variables, and Mann–Whitney U-tests or Kruskal–Wallis tests for non-normally distributed data. Categorical variables were compared using Chi-square tests. The relationships between dementia knowledge and PCA-derived component scores for supportive care practices were assessed using Pearson or Spearman correlation coefficients as appropriate. A *p*-value of less than 0.05 (two-tailed) was considered statistically significant for all tests.

## Results

A total of 136 family carers participated in this study. This section presents their sociodemographic backgrounds and caregiving profiles, which help contextualize their experiences and perspectives regarding dementia care. Table 1 summarizes the demographic and caregiving characteristics of 136 family carers of persons with dementia. Most carers were female (79.4%) with a mean age of 54.3 years, predominantly Buddhist, and over half were married. The majority were daughters (42.6%) or wives (14.7%) of the patients. Nearly three-quarters had at least a bachelor's degree. The median duration of caregiving was 2 years, with most providing care in the same household. About two-thirds shared caregiving responsibilities with others, but only a quarter received external organizational support. The most common reasons for hospitalization of persons with dementia were mood or behavioral symptoms, infections, and gastrointestinal issues. Following discharge, the perceived health outcomes of persons with dementia were reported as unchanged (44.1%), improved (36.0%), or worsened (19.9%) by carers.

**Table 1:** Demographic characteristics and caregiving profile of family carers (*N* = 136).

**Variable**	**Category**	***N* (%)**
Gender	Male	28 (20.6)
	Female	108 (79.4)
Age (years), Mean (SD)	54.3 (12.3)
Religion	Buddhism	129 (94.9)
	Others	7 (5.1)
Marital status	Single	37 (27.2)
	Partnered	2 (1.5)
	Married	78 (57.4)
	Separated	4 (2.9)
	Divorced	9 (6.6)
	Widowed	5 (3.7)
	Prefer not to say	1 (0.7)
Relationship to person with dementia	Wife	20 (14.7)
	Husband	4 (2.9)
	Daughter	58 (42.6)
	Son	16 (11.8)
	Father/Mother	15 (11.0)
	Siblings (same parents)	4 (2.9)
	Others (e.g., nanny, caregiver, daughter-in-law)	18 (13.2)
	Prefer not to say	1 (0.7)
Highest education attained	Primary school	7 (5.1)
	Lower secondary	6 (4.4)
	Upper secondary	14 (10.3)
	Diploma/associate degree	11 (8.1)
	Bachelor's degree	51 (37.5)
	Higher than a bachelor's degree	45 (33.1)
	Prefer not to say	2 (1.5)
Current employment status	Working	75 (55.6)
	Previously worked, now not working	58 (43.0)
	Never worked	2 (1.5)
Left Job to Become Carer (*n =* 58)	Yes	21 (36.2)
	No	37 (63.8)
Years Since Relative Diagnosed with Dementia, Median (IQR)		4.6 (2.0–8.0)
Years Providing Care, Median (IQR)		2.0 (1.0–5.0)
Place of Care	Same house as carer	105 (77.2)
	Different house	27 (19.9)
	Private nursing home	3 (2.2)
	Others	1 (0.7)
Co-caregiver (equal responsibility)	No	48 (35.3)
	Yes	88 (64.7)
Number of Co-Caregivers, Median (IQR)	2.0 (1.0–2.0)
Received help from organization/group	No	90 (66.2)
Yes	34 (25.0)
Prefer not to say	12 (8.8)
Type of help received^*^	Training/Education	12 (35.3)
	Financial support	8 (23.5)
	Joined a support group	12 (35.3)
	Arranged a professional caregiver	4 (11.8)
	Others (e.g., media, etc.)	9 (26.5)
	No. of hospital admissions after dementia diagnosis, median (IQR); range	2.0 (1.0–3.0); 1–20
Duration of most recent admission (days), median (IQR); range		5.0 (3.0–11.0); 2–11
Primary reason for last hospitalization	Mood/behavioral problems	31 (22.8)
	Pneumonia	16 (11.8)
	Urinary tract infection	13 (9.6)
	Other infections	12 (8.8)
	Gastrointestinal issues	15 (11.0)
	Fracture	7 (5.1)
	Accident	5 (3.7)
	Brain bleed/Stroke	9 (6.6)
	Refusal to eat/drink	4 (2.9)
	Cancer	1 (0.7)
	Cardiovascular/Coronary disease	9 (6.6)
	Electrolyte imbalance	2 (1.5)
	Arthritis	4 (2.9)
	Others (e.g., seizure, eye, skin, hepatitis, etc.)	17 (12.5)
Overall, health after discharge	Improved	49 (36.0)
	Worsened	27 (19.9)
	The same	60 (44.1)

^*^Multiple responses allowed.

Table 2 presents carers' perceptions of the implementation and sufficiency of dementia-friendly Practices in the Hospitals. While family involvement in care and decision-making was generally reported as sufficient (over 80%), several key dementia-friendly measures were inconsistently implemented. Nearly half of respondents indicated the absence of standardized dementia identification protocols (47.1%) and limited communication of discharge plans with community providers (45.6%). Hospital staff frequently succeeded in providing regular health updates (75.7%), assigning consistent care teams (69.9%), and minimizing unnecessary moves (66.9%). However, only about half of carers felt that care was adequately personalized, with systematic documentation of preferences and individualized activities often lacking. Access to palliative care and opportunities for advance care planning were also insufficient for a substantial proportion of families. These results highlight strengths in family engagement but point to significant gaps in structured dementia care processes and continuity planning within Thai hospital settings.

**Table 2:** Implementation and Sufficiency of Dementia-Friendly Practices (ISDP) in Thai hospitals (*N* = 136).

	**ISDP item**	**No *n* (%)**	**Yes, but insufficient, *n* (%)**	**Yes, and sufficient *n* (%)**
1	Establish standardized protocols to facilitate the identification of patients with dementia, thereby prompting staff to deliver dementia-specific care.	64 (47.1)	18 (13.2)	54 (39.7)
2	Allocate additional time for healthcare professionals to communicate with and understand patients with dementia and their families compared to the general patient population.	23 (16.9)	36 (26.5)	77 (56.6)
3	Ensure that medical personnel regularly introduce themselves, clarify ward locations, and provide clear explanations for hospitalization to mitigate patient confusion and anxiety.	17 (12.5)	22 (16.2)	97 (71.3)
4	Limit unnecessary transfers or room changes for patients with dementia to enhance their stability and orientation.	33 (24.3)	12 (8.8)	91 (66.9)
5	Coordinate staff scheduling to promote consistency and familiarity in the caregiving team for individuals with dementia.	19 (14.0)	21 (15.4)	95 (69.9)
6	Support the personalisation of patients' environments to foster comfort and reduce distress.	34 (25.0)	13 (9.6)	89 (65.4)
7	Engage families in discussions regarding the patient's preferences, interests, and aversions to inform individualized care planning.	50 (36.8)	17 (12.5)	69 (50.7)
8	Systematically document and disseminate key personal information and behavioral tendencies among the multidisciplinary care team.	45 (33.1)	23 (16.9)	68 (50.0)
9	Facilitate patient access to individualized, meaningful activities such as music, television, and reading to enhance wellbeing.	41 (30.1)	18 (13.2)	77 (56.6)
10	Encourage and accommodate the participation of family members willing to assist with aspects of care, including feeding, hydration, and personal hygiene.	8 (5.9)	14 (10.3)	114 (83.8)
11	Involve family members in clinical decision-making processes throughout hospitalization.	7 (5.1)	10 (7.4)	118 (86.8)
12	Provide regular and transparent updates to families regarding the patient's health status and treatment interventions.	10 (7.4)	22 (16.2)	103 (75.7)
13	Ensure availability of a palliative care team to support comprehensive assessment and advanced care planning.	45 (33.1)	19 (14.0)	72 (52.9)
14	Promote open and ongoing discussions around advanced care planning and end-of-life decision-making for patients with dementia.	55 (40.4)	18 (13.2)	63 (46.3)
15	Incorporate families into discharge planning and the formulation of post-discharge care strategies.	34 (25.0)	19 (14.0)	83 (61.0)
16	Communicate discharge care plans with relevant local healthcare providers and residential facilities to support seamless continuity of care.	62 (45.6)	18 (13.2)	56 (41.2)

An additional observation from Table 2 is the marked discrepancy in responses for several ISDP items, such as standardized identification, documentation practices, provision of palliative care, advance care planning, and discharge communication, where nearly equal proportions of carers reported “Yes and sufficient” and “No,” with relatively few selecting “Yes but insufficient.” This polarized response pattern suggests a high degree of variability in carers' experiences of these dementia-friendly practices across hospital sites.

To explore the underlying construct validity of the ISDP questionnaire, we conducted a principal component analysis (PCA) with varimax rotation using all 16 ISDP items. This statistical approach allowed identification of item groupings representing key domains of dementia-friendly hospital practice as perceived by carers. Table 3 presents the factor structure of the ‘Implementation and Sufficiency of Dementia-Friendly Practices' (ISDP) questionnaire, as determined by principal component analysis (PCA) of its 16 items. This table demonstrates how specific ISDP items loaded onto four distinct factors following varimax rotation. Four main factors were identified: (1) Person- and Family-Centered Advance Care Planning; (2) Dementia Identification and Supportive Care; (3) Environmental Stability and Meaningful Engagement; and (4) Family Involvement and Communication. The pattern of factor loadings is shown below.

**Table 3:** Factor Structure of Implementation and Sufficiency of Dementia-Friendly Practices (ISDP).

**Item no**.	**ISDP item**	**Person- and family-centered advance care planning**	**Dementia identification and supportive care**	**Environmental stability and meaningful engagement**	**Family involvement and communication**
14	Discuss advanced and end-of-life care.	**0.859**	0.163		
13	Ensure palliative care access.	**0.795**		0.101	0.289
15	Include families in discharge plans.	**0.726**	0.253		0.142
8	Share patient info with the care team	**0.600**	0.25	0.526	0.132
7	Involve families in care planning.	**0.581**	0.122	0.531	0.18
16	Share discharge info with community providers.	**0.574**	0.367		
2	Allow more time for patient-family communication	0.126	**0.726**	0.285	
3	Staff introduce and explain clearly	0.142	**0.724**	0.168	0.289
1	Standardize dementia identification.	0.263	**0.595**	0.188	
6	Personalize patient environments.	0.191	**0.562**		
4	Minimize room transfers for stability.		0.194	**0.757**	
5	Keep consistent caregiving staff.	0.156	0.384	**0.560**	0.203
9	Offer personalized, meaningful activities.	0.345		**0.520**	0.39
10	Support family help with daily care.	0.138	−0.1	0.219	**0.768**
12	Keep families regularly informed.	0.273	0.291		**0.676**
11	Include family in care decisions.		0.334		**0.651**

Bold values indicate primary factor loadings ≥ 0.50, representing items that load most strongly on each respective factor.

Table 4 presents dementia knowledge levels among carers, as assessed by the DKAS-Thai, concerning demographic characteristics. The overall mean DKAS-Thai score was 22.6 (SD = 7.0). There were no statistically significant differences in dementia knowledge by gender (*p* = 0.357), religion (*p* = 0.975), or marital status (*p* = 0.918). For education level, carers with more than a bachelor's degree had higher mean scores, but this difference was not significant (*p* = 0.146). Similarly, age showed no significant correlation with dementia knowledge (*r* = −0.061, *p* = 0.487). These findings indicate that dementia knowledge among family carers was moderate and did not significantly vary across demographic subgroups.

**Table 4:** Levels of Dementia Knowledge (DKAS-Thai) and perceived Implementation of Dementia-Friendly Practices (ISDP) according to participants' characteristics.

**Demographic characteristics**	**DKAS-Thai score**	**ISDP**			
		**Person- and family-centered advance care planning**	**Dementia identification and supportive care**	**Environmental stability and meaningful engagement**	**Family involvement and communication**
**All participants** (*n =* 136)	22.6 (7.0)	12.88 (4.2)	9.32 (2.3)	7.24 (1.8)	8.29 (1.2)
**Gender**					
Male (*n =* 28)	21.5 (6.9)	13.25 (3.8)	9.46 (2.2)	7.29 (1.9)	8.25 (1.2)
Female (*n =* 108)	22.9 (7.1)	12.79 (4.2)	9.28 (2.3)	7.23 (1.8)	8.30 (1.2)
**Religion: mean (SD)**					
Buddhism (*n =* 129)	22.6 (7.1)	12.95 (4.1)	9.33 (2.3)	7.25 (1.8)	8.31(1.2)
Others (*n =* 7)	22.7 (6.3)	11.71 (4.3)	9.00 (2.4)	7.14 (2.0)	7.86(1.2)
**Marital status**					
Married/partnered (*n =* 84)	22.7 (6.9)	13.01 (4.2)	9.45 (2.4)	7.35 (1.8)	8.49 (1.0)
Single/separate/divorced/widowed (*n =* 52)	22.6 (7.2)	12.71 (4.1)	9.14 (2.3)	7.11 (1.9)	8.02 (1.5)^*^
**Education**					
< High school (*n =* 15)	20.9 (5.7)	14.46 (3.1)	9.31 (2.4)	7.54 (1.7)	8.85 (0.6)
High school/Certificate (*n =* 25)	21.9 (7.4)	13.40 (4.2)	9.16 (2.4)	7.40 (1.8)	8.40 (1.1)
Bachelor's degree (*n =* 51)	21.8 (6.2)	12.49 (4.4)	8.92 (2.4)	7.02 (1.8)	8.18 (1.4)
> Bachelor's degree (*n =* 45)	24.6 (7.9)	12.60 (4.0)	9.83 (2.3)	7.33 (1.7)	8.20 (1.2)
Others					

Values are presented as mean scores, with standard deviations (SD) shown in parentheses. DKAS-Thai scores reflect carer knowledge about dementia. ISDP subscale scores represent perceived implementation and sufficiency of dementia-friendly practices across four domains: Person- and Family-Centered Advance Care Planning, Dementia Identification and Supportive Care, Environmental Stability and Meaningful Engagement, and Family Involvement and Communication. Statistical comparisons were conducted using Analysis of Variance (ANOVA), ^*^p < 0.05.

Regarding the correlation between the total Dementia Knowledge Assessment Scale (DKAS) scores and the factors of the ISDP. The results showed no significant correlations between dementia knowledge and any of the factors: Person- and Family-Centered Care: *r* = −0.025, *p* = 0.769, Dementia Identification and Supportive Care: *r* = −0.166, *p* = 0.053, Environmental Stability and Engagement: *r* = −0.018, *p* = 0.838, Family Involvement and Communication: *r* = −0.047, *p* = 0.591. All correlation coefficients were close to zero and not statistically significant (*p* > 0.05), indicating that overall dementia knowledge, as measured by the DKAS, was not associated with attitudes or practices across the identified dementia care domains among participants. Additional analyses were conducted to explore potential non-linear relationships between carer dementia knowledge (DKAS scores) and perceptions of dementia-friendly practices (ISDP). Curve estimation analyses, including inspection of scatterplots, showed that an inverse model provided the closest statistical fit; however, this association did not reach significance (*F* = 3.60, *p* = 0.060, *R*^2^ = 0.027). Therefore, no significant linear or non-linear association was identified between DKAS and ISDP scores in this sample.

## Discussion

This multi-center study, conducted in four major university hospitals across Thailand, provides valuable insights into the demographics, knowledge, and hospital care experiences of family carers for older adults with dementia. By focusing on tertiary care, university hospitals equipped with advanced geriatric and neurological services, our findings offer a distinctive perspective that reflects both the opportunities and challenges inherent in delivering dementia care within high-resource academic environments.

In this study, we found that many family carers still had unclear or incomplete knowledge about dementia, despite receiving care from leading university hospitals. The Dementia Knowledge Assessment Scale—Thai version (DKAS-Thai) showed that, while some aspects, such as recognizing memory loss as a sign of dementia, were relatively well understood, other knowledge gaps persisted. For example, misconceptions about the reversibility of dementia and the appropriate management of behavioral symptoms were still common (23).

Our data revealed that carer knowledge, as measured by the DKAS-Thai, varied depending on carer education level, prior exposure to dementia, and relationship to the patient. Even among carers accessing university hospitals, areas such as dementia symptom recognition and care pathway navigation remained suboptimal for many respondents. This indicates that while university hospitals play a leading role in piloting educational interventions, more structured and accessible family education programs are needed to ensure carers are adequately informed and empowered (24, 25).

Although we found no statistically significant linear or non-linear association between carer dementia knowledge and perceptions of dementia-friendly practices, it is possible that site-specific differences in dementia care protocols and practices introduced heterogeneity, potentially masking associations within individual hospitals when data are analyzed at the aggregate (group) level. These findings suggest that factors beyond carer knowledge, such as organizational context, care environment, and cultural influences, may play a more substantial role in shaping carers' perceptions of hospital dementia care. Future research using stratified or multilevel analyses is recommended to further explore whether site- or context-specific factors influence these relationships (26). Furthermore, our results showed that the implementation and sufficiency of dementia-friendly hospital care practices were inconsistent. Despite the presence of specialists and better resources in university hospitals, carers reported moderate levels of satisfaction regarding communication with staff, environmental adaptations, and their involvement in care planning. The Implementation and Sufficiency of Dementia-Friendly Practices (ISDP) scores highlighted both the progress and the remaining limitations in these high-resource settings. As shown in the results, the response patterns were polarized, suggesting a high degree of variability in carers' experiences of these dementia-friendly practices across hospital sites. The reasons for these discrepancies are likely to reflect site-level variation in dementia care protocols, differences in carers' expectations and awareness, and potential limitations in the ISDP response categories. Some hospitals may have well-established procedures, while others lack them entirely, contributing to the polarized “all-or-none” pattern. Furthermore, carers with less knowledge may not recognize partial implementation, and cultural factors could affect the willingness to express moderate dissatisfaction (27, 28). Multi-center sampling may also have amplified these site-specific perceptions.

The recruitment method used in this study, through outpatient follow-up clinics post-discharge, underscored the continued engagement of family carers with multidisciplinary hospital teams. However, the study also revealed that such ongoing relationships did not always translate into smooth communication or comprehensive support for carers. Greater inclusion of family carers in discharge planning and ongoing multidisciplinary case discussions could help address these gaps (13, 24).

Our findings align with previous Thai and international studies reporting on the challenges that carers encounter in hospital settings (11, 13). Barriers such as a lack of staff training in dementia care, time pressure, and the division of responsibilities between staff and family were commonly reported. Importantly, our research points to the discrepancy between policy ideals—such as the adoption of dementia-friendly, person-centered care pathways—and the realities experienced by family carers in university hospital settings.

Notably, the broader Thai cultural context significantly shapes these experiences. Family carers often assume caregiving out of filial obligation and cultural expectations, sometimes at the cost of their own emotional wellbeing. While the university hospital environment may provide advanced knowledge and available specialists, cultural stigma about dementia and expectations of family self-sufficiency continue to influence both the care process and the willingness to seek formal support (21, 29). These findings strengthen the case for culturally sensitive policy-making and program development within academic medical centers.

Another important issue is communication between hospital staff and carers. Many carers in this study noted that they were not always given clear, practical advice or invited to participate meaningfully in care decisions. This lack of effective communication may hinder effective transitional care and discharge planning, potentially impacting both patient outcomes and carer stress levels (30).

These results are particularly relevant given that university hospitals often serve as benchmarks for standard-setters in training, care quality, and policy for other hospitals across Thailand. The study's multi-regional university hospital sample strengthens the generalisability of the insights, while highlighting opportunities for further improvement and national policy development.

This study has some limitations. The sample was restricted to family carers attending outpatient clinics at four university hospitals, possibly excluding less involved or more vulnerable family carers. In addition, cross-sectional design limits causal inference, and some bias may remain due to self-reporting and recall.

In summary, by focusing explicitly on university hospitals and integrating empirical findings with cultural context, our study underscores the urgency and opportunity for advancing family-centered, dementia-friendly care in Thailand's leading healthcare institutions. Structured family education, multidisciplinary engagement, and culturally responsive policy adjustments should be prioritized to close the remaining gaps in support for carers of people living with dementia (21, 25). These lessons are highly relevant not only for university hospitals, but also for informing the ongoing development of dementia care in regional and community healthcare settings throughout the country.

## Clinical implications

The findings from this study highlight several actionable clinical implications for improving dementia care in Thai general hospitals. Despite notable strengths in family engagement and communication, key gaps were evident in areas such as early dementia identification, structured advanced care planning, interdepartmental information sharing, and continuity of care post-discharge. Addressing these gaps requires system-level interventions, including the development and enforcement of standardized dementia screening protocols, routine involvement of families in discharge planning, greater access to palliative care teams, and structured, multidisciplinary care pathways. Furthermore, results indicate that increasing staff knowledge alone is insufficient; hands-on training, supportive supervision, and fostering a dementia-friendly hospital culture are critical to translating knowledge into high-quality, person-centered practice.

## Strengths and limitations

A major strength of this study is its focus on the perspectives of family carers, who provide unique and valuable insights into the real-world delivery of dementia care across multiple hospital settings in Thailand. The use of a factor analysis approach also enabled a nuanced assessment of dementia care domains, supporting targeted recommendations. However, several limitations must be acknowledged. The study relied on self-reported data from carers, which may be subject to response bias or recall inaccuracies. The sample was limited to select general hospitals and may not be representative of all Thai healthcare settings, particularly rural or resource-limited areas. Additionally, the cross-sectional design precludes any causal inference between variables such as staff knowledge and care quality.

## Future research

Future studies should explore the experiences and perspectives of multiple stakeholders, including hospital staff, patients, and community care providers, to build a more comprehensive understanding of system-level barriers and enablers. Longitudinal or intervention studies could help clarify the mechanisms by which knowledge, training, staff attitudes, system-level support, and organizational factors impact care quality and outcomes. There is also a need to develop, implement, and evaluate structured protocols for dementia identification, information sharing, and advanced care planning tailored to Thai hospital contexts. Finally, research on scalable models for integrating hospitals and community-based dementia support could inform national policy and practice.

## Conclusions

This study provides the first multi-center assessment of the knowledge and hospital care experiences of family carers for older adults with dementia in university hospitals across Thailand. The results show that, while university hospitals are well-resourced and offer access to specialist care, significant variability remains in the knowledge levels of family carers and in the consistent implementation of dementia-friendly practices. Carer knowledge about dementia was generally moderate, with persistent gaps in understanding symptoms, care strategies, and disease progression, even within this specialized setting. The study also found that the perceived implementation and sufficiency of dementia-friendly practices varied, with carers commonly reporting limited involvement in care decision-making and variable communication with hospital staff.

A positive association was observed between higher carer knowledge and the perceived adequacy of dementia care practices, highlighting the potential impact of carer education on care quality and experiences within university hospital settings. The findings additionally underscore the influence of cultural expectations on caregiving roles and the need for culturally sensitive approaches.

## Data Availability

The raw data supporting the conclusions of this article will be made available by the authors, without undue reservation.
